# In vivo role of *Candida albicans β*-hexosaminidase (*HEX1*) in carbon scavenging

**DOI:** 10.1002/mbo3.274

**Published:** 2015-07-14

**Authors:** Deepa Ruhela, Mohan Kamthan, Paramita Saha, Subeer S Majumdar, Kasturi Datta, Malik Zainul Abdin, Asis Datta

**Affiliations:** 1 National Institute of Plant Genome Research, Aruna Asaf Ali MargNew Delhi, 110067, India; 2 Indian Institute of Toxicology ResearchLucknow, 226001, India; 3 Biochemistry Laboratory, School of Environmental Sciences, Jawaharlal Nehru UniversityNew Delhi, 110067, India; 4 Division of Cellular Endocrinology, National Institute of ImmunologyNew Delhi, India; 5 Department of Biotechnology, Faculty of Science, Jamia Hamdard UniversityNew Delhi, 110062, India

**Keywords:** Candida, hexosaminidase, hyaluronic acid, *N*-acetylglucosamine

## Abstract

The capability to utilize of *N*-acetylglucosamine (GlcNAc) as a carbon source is an important virulence attribute of *Candida albicans*. But there is a lack of information about the in vivo source of GlcNAc for the pathogen within the host environment. Here, we have characterized the GlcNAc-inducible *β*-hexosaminidase gene (*HEX1*) of *C. albicans* showing a role in carbon scavenging. In contrast to earlier studies, we have reported *HEX1* to be a nonessential gene as shown by homozygous trisomy test. Virulence study in the systemic mouse murine model showed that *Δhex1* strain is significantly less virulent in comparison to the wild-type strain. Moreover, *Δhex1* strain also showed a higher susceptibility to peritoneal macrophages. In an attempt to determine possible substrates of Hex1, hyaluronic acid (HA) was treated with purified Hex1 enzyme. A significant release of GlcNAc was observed by gas chromatography-mass spectrometry analysis analysis suggesting HA degradation. Interestingly, immunohistochemistry analysis showed significant accumulation of HA in the mice kidney infected with the wild-type strain of *C. albicans*. Northern blot analysis showed that *C. albicans HEX1* is expressed during mice renal colonization. Thus, *C. albicans* can obtain GlcNAc during organ colonization by secreting Hex1 via degradation of host HA.

## Introduction

*Candida albicans* is a common opportunistic human pathogen causing a disease called Candidiasis or Candidosis. It is the leading cause of fungal nosocomial blood stream infection with a high mortality rate in immunocompromised patients. *Candida albicans* can cause cutaneous, mucosal, and systemic infections and is normally engulfed by macrophages and neutrophils.

*Candida albicans* displays significant metabolic flexibility to assimilate available nutrients in diverse niches that it inhabits within its human host. Studies have shown that *C. albicans* phagocytosed by macrophages and neutrophils uses glyoxylate and gluconeogenesis pathways to assimilate carbon for growth (Barelle et al. 2006). In advanced stages of infection when the fungus causes disseminated infections in host tissues such as kidneys, glycolytic pathway is used for the assimilation of carbon. One of the important properties of several diverse human pathogens such as *C. albicans* (Singh et al. 2001), *Vibrio cholarae* (Ghosh et al. 2011), and *Leishmania major* (Naderer et al. 2010) is utilization of amino-sugars like *N*-acetyl-d-glucosamine (GlcNAc) as the sole carbon source. GlcNAc metabolic pathway mutants of these pathogens have significantly reduced virulence in mouse model. Thus, GlcNAc is utilized as in-vivo carbon source by these pathogens. In *C. albicans* GlcNAc induces the genes of the GlcNAc catabolic pathway (Singh and Datta 1979; Kumar et al. 2000). This pathway consists of four enzymes – namely, GlcNAc transporter (Ngt1), GlcNAc kinase (Hxk1), GlcNAc-6-phosphate deacetylase (Dac1), and GlcN-6-phosphate deaminase (Nag1), all of which act sequentially on GlcNAc to generate fructose-6-phosphate which is fed into the glycolytic pathway. GlcNAc is a monosaccharide derivative of glucose. In *C. albicans*, GlcNAc can regulate the expression of genes involved in metabolism, morphogenesis (Shepherd et al. 1980), and switching (Huang et al. 2010).

Although utilization of GlcNAc by human pathogens as in vivo carbon source is well established but source of GlcNAc within the body is not explored. Within the gastrointestinal tract GlcNAc could be released by bacteria. Another possible source of GlcNAc could be glycosaminoglycan (GAGs) present in the extracellular matrix (ECM). Hyaluronic acid (HA) is one of the members of GAG family present in the ECM. It is a large polysaccharide composed of repeating disaccharide units of glucuronic acid and GlcNAc. HA is present in abundant amount in the ECM of vertebrate tissues. HA is required for maintaining the elastoviscosity of liquid connective tissues, tissue hydration, it also has a role in receptor-mediated cell detachment, mitosis, and inflammation (Balazs et al. 1986; Toole et al. 2002; Turley et al. 2002; Hascall et al. 2004). The metabolism of HA is a highly controlled process due to its wide distribution and diverse role in tissues. In vertebrates, HA is synthesized by integral membrane protein known as hyaluronan synthase and extruded via ATP-binding cassette (ABC) transporter through the cell membrane into the extracellular space. Within the ECM, HA is first catabolised into smaller fragments by an endoglycosidase known as hyaluronidase. These small fragments are internalized by endosomes via a receptor-mediated interaction. After internalization, the endosome matures into lysosome, where other hyaluronidase further cleaves these fragments into short oligosaccharides. These oligosaccharides are further digested by exoglycosidases like *β*-glucuronidases and *β*-hexosaminidase. Hexosaminidase mutations lead to faulty hydrolysis of certain sphingolipids, which accumulate in lysosomes within the brain, resulting in the lipid storage disorders like Tay–Sachs and Sandhoff. Here, we explored the role of *β*-hexosaminidase of *C. albicans* in carbon scavenging via breakdown of HA.

## Experimental Procedures

### Mice

Female BALB/c mouse weighing 18–20 g were obtained as pathogen-free mice from the animal house of Jawaharlal Nehru University (JNU) New Delhi, India. The use of mice was duly approved by the Institutional Animal Ethic Committee (IAEC) of JNU, Registration No. 19/1999 (CPCSEA; Committee for the purpose of control and supervision of experiments on animals). Approval code was VO/AH/IAEC/84/53. All housing and experimental procedures were conducted under the guidelines of the JNU animal care.

### Strains, media and growth conditions

*Candida albicans* wild-type strain SC5314 was used in this study. For homozygous trisomy (HT) test, BWP17 (*∆ura3/∆arg4/∆his1*) strain was used. *Candida albicans* strains were routinely cultured in YPD medium (Yeast extract-1%, Peptone-2%, and Dextrose-2%) at 37°C with 200 rpm shaking and induced by GlcNAc (Sigma-aldrich India, A8625) in SN medium (0.67% yeast nitrogen base without amino acid and 2% GlcNAc) at 37°C. For growth on plates, 1.5% agar was added to the medium. For auxotrophic strains uridine 80 mg/L, 20 mg/L l-histidine, and 40 mg/L l-arginine were added. *Escherichia coli* strains were cultured in Luria-Bertani broth or agar plates containing 50 μg/mL Ampicillin.

### HT test

For HT test (Enloe et al. 2000) plasmid, pBME101 carrying *Ura3′-ARG4-Ura3′* cassette, was generously gifted by Aaron P. Mitchell. This plasmid was digested with *PvuII* to release the 4.217 kb fragment carrying Ura3′*-ARG4-Ura3′*. Simultaneously, 2.7 kb of *HEX1* orf with 1 kb upstream and downstream fragments was PCR amplified from genomic DNA using TTGAATGTCAAGACTGTTGTCC and AACTTCCGTTCCCTTTTGAGC as forward and reverse primers, respectively, and cloned to generate pA1. pA1 was digested with *Hinc II* to remove 1 kb fragment from 2.7 kb insert leaving 1.3 and 0.475 kb regions of *HEX1*. The 4.217 kb *Ura3′-ARG4-Ura3′* cassette was then ligated to pA1 to create the plasmid pUAU. The 8.96 kb recombinant pUAU plasmid was identified by its difference in migration on a 0.8% agarose gel. For generation of first allele knockouts 5 *μ*g of recombinant plasmid, pUAU was digested with *NotI* and *Nco1* to release the 5.96 kb disruption cassette (*HEX1:Ura3′-ARG4-Ura3′:HEX1*). Cassette was transformed into BWP17 (*∆ura3/∆arg4/∆his1*) strain (generously gifted by Aaron P. Mitchell) by electroporation method. Rest of the steps were followed as described elsewhere (Enloe et al. 2000).

### Disruption of *HEX1* by SAT flipper strategy

Null mutant was also generated by “SAT-Flipper Strategy” (Reuss et al. 2004) in the wild-type strain of *C. albicans* (Data S1). For the selection of nourseothricin-resistant (NouR) transformants, 200 *μ*g/mL of nourseothricin (Werner Bioagents, Jena, Germany) was added to YPD agar plates. To obtain nourseothricin-sensitive (NouS) derivatives, transformants were grown for 6 h in YPD medium without selective pressure and 150–250 cells were then spread on YPD plates containing 25 *μ*g/mL of nourseothricin for 2 days at 30°C. To verify that any mutant phenotypes were indeed caused by deletion of the target gene, a functional *HEX1* copy was reinserted into the homozygous hex1 mutant (Data S1).

### Southern blotting

For Southern blotting, genomic DNA was extracted from cells grown in YPD or SD media. A total of 5 *μ*g of DNA was digested with *SpeI* and *EcoRV* for HT test. Digested DNA was resolved by agarose gel electrophoresis on 1% agarose gels, before transferring (Sambrook et al. 1989) them to positively charged nylon membranes (NEN Research Products Ltd. USA) by capillary transfer. For screening HT test transformants hybridizations were performed with *α*P^32^ radiolabeled *Spe1* and *EcoRV* digested fragment of *HEX1*.

### Quantitative real time polymerase chain reaction (qRT-PCR)

Cells were grown in SD medium (0.67% yeast nitrogen base without amino acids and 2% glucose) and induced by GlcNAc in SN medium (0.67% yeast nitrogen base without amino acids and 2% GlcNAc) at 37°C and qRT-PCR was performed as described elsewhere (Kamthan et al. 2013).The following primers designed with Primer Express (V-3.0) software (Applied Biosystems, California, USA) were used for qPCR analysis. ACT1-F5′-GACAATTTCTCTTTCAGCACTAGTAGTGA-3′ and ACT1-R5′-GCTGGTAGA GACTTGACCAACCA-3′; HEX1-F5′-CTTGGAGCGGGAACAAGGAT-3′ and HEX1-R5′-CACAA GCGTGTGGATTGAGC-3′; GIG1-F5′-ACGTTAATGCCTCAACCATCG-3′ and GIG1-R5′-TGCT GTCGTGATCGAGCAAA-3′; HXK1-F5′-TGTGTCGTCGCAAGAATCCA-3′ and HXK1-R5′-TCGA TGCAGATACCGCAGAC-3′; DAC1- F5′-CCACAGCCACATCACCGTAA-3′ and DAC1-R5′-A TCGACGGGTCAACATGTACAC-3′; NAG1-F5′-CAACGAAGCGGGATCATCA-3′ and NAG1-R5′T TCCCGAAAAACCTGCAGTT-3′.

### Virulence study

BALB/c (female) mice weighing between 18–20 g (8–10 weeks old) were used to test the virulence of different strains. The strains SC5314 (Wild type), *hex1/hex1*, and *hex1/hex1/HEX1* (*h/h/HEX1*) were grown on YPD, at 37°C for 48 h. The cells were suspended in phosphate-buffered saline (pH 7.5) to desired cell density. 1 × 10^7^ cells were intravenously injected into mice via the lateral tail vein. Control mice were injected with 200 *μ*L Phosphate buffer saline (PBS) as a vehicle control. Group of five mice per *C. albicans* strain were inoculated for a morbidity assay. The course of infection was monitored for as long as 45 days. For histopathology studies, another group of three mice each were similarly injected and sacrificed after 48 h post injection. Kidneys removed postinfection were fixed in 10% formaldehyde–PBS, sectioned in paraffin blocks, and stained with periodic acid-Schiff reagent (PAS) and haematoxylin–eosin (H&E) stain. Examination of the sections was performed under a light microscope, and photographs were taken with a Nikon, USA camera fitted to the microscope. For studies in mouse peritoneal cavity, Freund's complete adjuvant (0.4 mL) was injected into the peritoneal cavity of female BALB/c mouse, weighing 17–22 g. Ten days after the injection, a significant abdomen enlargement was observed due to large amount of exudates in the peritoneal cavity. Further, 2 × 10^8^ late log phase cells of each strain in 200 mL of PBS was injected into enlarged peritoneal cavity (three mice for each strain were used). Twenty-four hours after the injection, 100 *μ*L of the peritoneal exudate was retrieved using a 23G hypodermic needle attached to a 1 mL syringe. Peritoneal exudates were smeared on microscopic glass slides and stained with PAS. Photographs were taken with a Leica digital camera, Germany attached to the microscope. A total of 10 *μ*L of peritoneal exudates was added to 190 *μ*L sterile MQ for lysing the macrophages and the solution was plated on YPD agar and incubated at 37°C for 3 days to determine the *C. albicans* colony forming units (CFU).

### In vitro degradation of HA by *Hex1*

HA (0.4 mg/mL) was digested with purified hexosaminidase protein (50 U/mL) or with both hyaluronidase (50 U/mL) and hexosaminidase (50 U/mL) or only with hyaluronidase (50 U/mL). Tubes were incubated at 37°C overnight followed by gas chromatography-mass spectrometry (GC/MS) analysis to estimate free GlcNAc.

### GC/MS analysis to detect free GlcNAc

Pretreated samples were dried in vacuo at 40°C. The dried residue was redissolved and derivatized for 90 min at 37°C in 80 *μ*L of 20 mg mL^−1^ methoxyamine hydrochloride in pyridine followed by 30-min treatment at 37°C with 80 *μ*L N-Methyl-N-(trimethylsilyl)trifluoroacetamide (MSTFA). Samples were diluted 10-folds in n-heptane and 1 *μ*L was injected in splitless mode. GC/MS analysis was performed on Shimadzu GC/MS-QP 2010 plus as described elsewhere (Roessner et al. 2000). The mass spectrometer was tuned according to the manufacturer's recommendations. GlcNAc 1 mg/mL was used as standard. 50 *μ*L Sorbose (1 mg/mL) and *α*-aminoisobutyric acid 80 *μ*L (1 mg/mL) was used as internal standard control. GC was performed on an Rtx5MS- 30 m column with 0.25-mm ID and 0.25 *μ*m df (Restek, USA). Concentration of metabolites was normalized with respect to the area of the internal standard peak.

### Northern blot analysis

Total RNA was isolated from the kidneys using Tripure reagent (Roche, USA). About 25 *μ*g of total RNA was separated on 1.5% agarose–formaldehyde gel and transferred onto a nylon membrane by capillary blotting (Sambrook et al. 1989). *α*P32-labeled *HEX1* (*C. albicans*) and *ACT1* gene (mouse) were used as a probe.

### HABP assay through immunohistochemistry

Kidney sections of the mice used for virulence study were also analyzed for presence of HA. Longitudinal sections of the kidneys were placed on the slide and gently warmed on flame for fixing. Slides were then treated with xylene from 5 min to overnight. Sections were rehydrated by exposing the slides to 96%, 80%, 70%, 50% alcohol for an interval of 5 min each. Slides were further kept in distilled water for 15 min followed by 30 min in 10 mmol/L sodium citrate pH 6.0 to activate an antigen at 39°C and washed with 1X PBS for 5 min (avoid drying). Sections were then overlaid with 3% Bovine serum albumin (BSA) in 1X PBS and incubated at 37°C for 1 h on a glass plate in humid chamber. The slides were washed with 1X PBS. Sections were further treated with primary antibody (biotinylated HA-binding protein [HABP]-biotin bovine) with 1:200 dilutions in 1.5% BSA in PBS at 37°C for 1 h in a humid chamber. After five washes of PBS for 5 min, each slide was treated with secondary antibody (Streptavidin-peroxidase) with 1:500 dilution of 1X PBS kept at 37°C for 1 h. The Slides were further washed with 1X PBS twice, 5 min each and overlaid with DAB (3,3′-diaminobenzidine) on the glass plate in dark humid chamber for color development. After washing with water to remove excess DAB, the sections were counterstained with Periodic acid–Schiff's reagent followed by H&E stain. Photographs were taken in Nikon 80i microscope at 10× and 60× magnifications. The entire experiment was repeated thrice.

### Purification of HA and separation on gradient polyacrylamide gel

HA purification was carried out from infected kidney. Around 100 mg kidney tissue of mice infected with wild-type, ∆hex1, or hex1/hex1/HEX1 revertant strains was treated with 200 *μ*L of 50 mmol/L sodium acetate (pH 6.0), containing 250 *μ*g/mL of proteinase K, 5 mmol/L Ethylenediaminetetraacetic acid (EDTA), and 5 mmol/L Cysteine for 5 h at 60°C. Proteinase K was inactivated by incubation in a boiling water bath for 10 min followed by centrifugation. Supernatant was collected and treated with 4 volumes of 1% cetylpyridinium chloride in 20 mmol/L NaCl for 1 h at room temperature, and the centrifuged at 13,000*g* for 15 min. After discarding the supernatant, the precipitate was washed with 1 mL of water, centrifuged again, and dissolved in 50 *μ*L of 4 mol/L guanidine-HCl. Furthermore, 900 *μ*L of ethanol was added and the tube was kept at −20°C for 1 h after which each sample was centrifuged and the precipitate was retained and dissolved in 50 *μ*L of 50 mmol/L sodium acetate (pH 6.7). Hyaluronan digestion was carried out using 50 *μ*g/mL of bovine testicular hyaluronidase (BTH) for 3 h at 37°C. Equal volumes of both undigested and BTH-digested products derived from kidneys were loaded onto a 5–20% gradient gel, along with BTH-digested and undigested pure polymeric HA acting as positive controls. The gel was then stained with 1% Alcian blue in 3% acetic acid, destained, and subsequently stained with silver nitrate.

### Estimation of GlcNAc by dimethylaminobenzaldehyde reagent

GlcNAc was estimated as described elsewhere (Elson and Morgan 1933) with slight modifications. About 100 mg kidney tissue was homogenized in 1 mL MQ. Further, 0.5 mL of 1.5 N Na_2_CO_3_ containing 4% acetyl acetone was added and heated to 100°C for 20 min. The solution was cooled and 3.5 mL of Ehrlich's reagent (10 mmol/L dimethylamino benzaldehyde in 50% ethanol and 6 N HCl, 0.5 mL) was added and incubated at room temp for 1 h. Hexosamine content was determined by measuring the Optical density (OD) at 520 nm. GlcNAc was used as standard.

## Results

### Phylogenetic and expression analysis of *C. albicans β*-hexosaminidase

**Figure 1:**
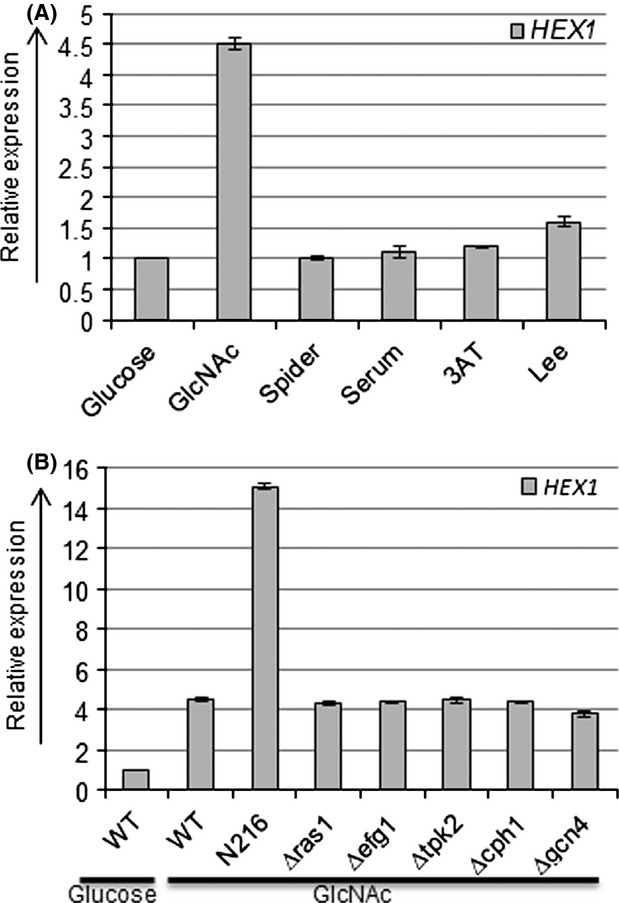
Expression analysis of *HEX1*. (A) Relative expression levels as determined by q-RT PCR analysis of the wild-type (SC5314) strain in presence of different media which promote morphogenesis in *Candida albicans*. (B) Relative expression levels as determined by q-RT PCR analysis to compare *HEX1* expression levels of wild-type (SC5314) strain and mutants of major signal transduction pathway that control morphogenesis (*∆efg1, ∆cph1, ∆gcn4, ∆ras1*, *and ∆tpk2*). Cells were grown at 37°C.

In *C.albicans CaHEX1* is a 1689 bp long gene encoding for a 562 amino acids protein belonging to glycol-hydro-20 superfamily and GH_20 HexA_HexB_-like hexosaminidase superfamily. Evolutionary relationship was established between Hex1 proteins of various species by phylogenetic tree construction using MEGA6 software (Fig. S1). Among the fungal homologs CaHex1 showed proximity to arthropod parasite *Beauveria bassiana* (98%) and *Cryptococcus neoformans* (68%), a pathogenic yeast causing infections in immunocompromised peoples. Interestingly, CaHex1 showed close proximity to *Arabidopsis thaliana* (98%) HexO2 which is a cell membrane associated protein. CaHex1 also showed close resemblance to *Trichoderma harzianum* (69%)*, Tribolium castaneum* (84%)*, Caenorhabditis elegans* (85%)*, Chinococcus granulosus* (83%)*,* and *Drosophila melanogaster* (87%). It was shown previously that *HEX1* is induced by GlcNAc in *C. albicans* (Sullivan et al. 1984). Since, GlcNAc is a strong inducer of morphogenesis in *C. albicans*, expression of *HEX1* was also analyzed under other hyphae-inducing conditions. qRT-PCR analysis showed that in comparison to GlcNAc, no significant expression of *HEX1* was observed under various hyphae-inducing conditions tested (Fig.1A). Furthermore, expression of *HEX1* was also analyzed in the mutants of major signal transduction pathway that controls morphogenesis in *C. albicans*. No significant difference in expression of *HEX1* was observed in the mutants (*∆efg1*, *∆cph1*, *∆gcn4*, *∆ras1*, and *∆tpk2*) as compared to that of wild type in presence of GlcNAc (Fig.1B). However, a threefold higher expression of *HEX1* was observed in GlcNAc metabolic pathway mutant N216 (*∆hxk1 ∆dac1 ∆nag1*) in comparison to the wild type under GlcNAc induction. The higher expression of *HEX1* in N216 mutant could be a result of GlcNAc accumulation in this mutant as it lacks the GlcNAc-metabolizing enzymes. It was also shown previously that higher level of GlcNAc-induced gene is observed in the triple (*∆hxk1 ∆dac1 ∆nag1*) GlcNAc metabolic pathway mutant (Naseem et al. 2011).

### Generation of hexosaminidase gene knockout in *C. albicans*

**Figure 2:**
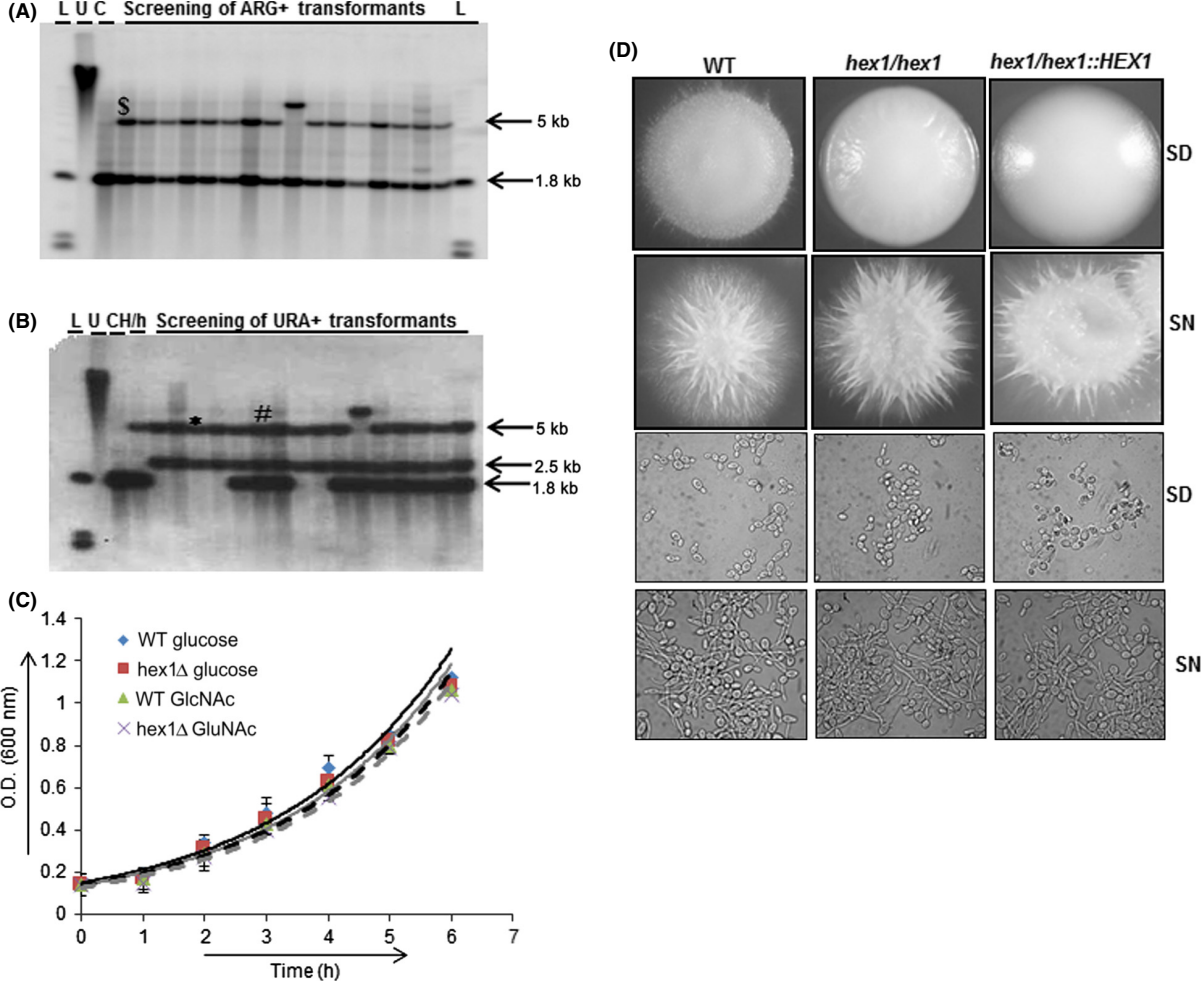
(A) Southern blot analysis to confirm the first allele knockouts for homozygous trisomy test. Autoradiogram showing the first allele knockout of *HEX1*. Strains with a 5 and 1.8 kb fragments are first allele knockouts ($) and wild-type strain only showed 1.8 kb band. (B) Autoradiogram to confirm the essentiality of *HEX1* through rare mitotic recombination. *Represents the double allele knockout strain (∆hex1-UAU) and #represents the *HEX1* triploid strain. For Southern blot analysis, *SpeI* – *EcoRV* enzymes were used to digest 5 *μ*g genomic DNA. *α*P^32^ labeled 1.8 kb *SpeI* – *EcoRV* digested *HEX1* was used as probe. L, 1 kb ladder; U, undigested DNA; C, BWP17 strain; H/h, 1st allele knockout. (C) Growth assays for indicated strains at 37°C in SD (2% glucose) represented by solid line and SN (2% GlcNAc) represented by dotted line. Error bars represent standard error between three separate experiments. (D) Comparative morphogenetic studies of wild-type, ∆*hex1*, and *hex1*/hex1/*Hex1* revertant strain on solid and liquid SD (2% glucose) and SN (2% GlcNAc) media. Plates were incubated at 37°C for 4 days. Cells were induced for 3 h in liquid media at 37°C.

Hexosaminidase mutants have been created earlier, through nontargeted approach using mutagenic agents (Jenkinson and Shepherd 1987). However, these knockouts could have multiple deletions which remain undetected. Moreover, through large-scale gene disruption by *UAU1* cassette in *C. albicans*, *HEX1* was shown to be an essential gene (Nobile and Mitchell 2009). In order to confirm the essentiality of *HEX1*, we performed specially designed single transformation-based gene function test called Homozygote Trisome test (Enloe et al. 2000). *Candida albicans* was transformed with the disruption cassette (*HEX1:Ura3′-ARG4-Ura3′:HEX1*). First, allele knockouts were confirmed by Southern blot analysis (Fig.2A). Positive first allele knockouts were allowed to grow in rich medium for generation of double allele knockouts (ARG4^+^ and URA^+^) through random gene conversion or mitotic events. Southern blot analysis revealed that independent ARG4+ URA+ segregants were either *hex1*::*UAU1*/*hex1*::*URA3*/*HEX1* triplication derivatives (these are derived from trisomy or translocation) or double knockout (∆hex1-UAU) *hex1*::*UAU1*/*hex1*::*URA3* (Fig.2B). Hence, this result contradicts the earlier result suggesting that *HEX1* is not an essential gene in *C. albicans*. But, due to ectopic expression of *URA3* at the *HEX1* locus these knockouts cannot be used for virulence and morphogenetic analysis (Cheng et al. 2003). So, for functional characterization of *C. albicans HEX1* knockouts were also generated by using the SAT1 flipper strategy in prototroph wild-type strain of *C. albicans* (Fig. S2A). Revertant strain (*hex1/hex1/HEX1*) with reintroduced *HEX1* allele at the native locus was also generated (Fig. S2B). The *hex1* mutant strains (*∆hex1* and *hex1/hex1/HEX1*) generated by SAT flipper strategy were then analyzed for their ability to grow in presence of GlcNAc. No significant difference was observed in the growth of *Δhex1* mutant as compared to wild-type strain in the presence of either glucose or GlcNAc (Fig.2C). No change was also observed in the GlcNAc-induced morphogenesis in the *Δhex1* strain and revertant strain (*hex1/hex1/HEX1*) as compared to wild type on both solid and liquid media (Fig.2D).

### Virulence of Δhex1 strain in mouse

**Figure 3:**
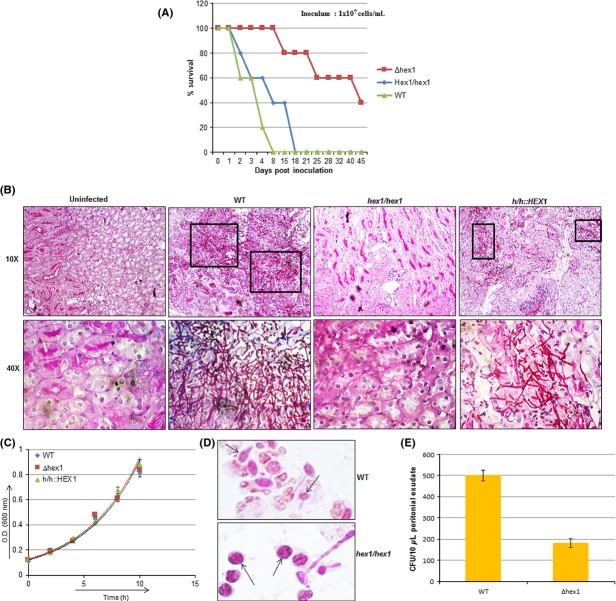
(A) Survival curve of mice injected with lethal dose (1 × 10^7^ cells/mL) of indicated *Candida albicans strains*. (B) Histopathology of infected mouse kidney with indicated strains. Mice were sacrificed 2 days post injection of lethal dose of *C. albicans* strains. Kidney sections were stained with Eosin and Hematoxylin. *Candida albicans* cells were visualized via PAS staining. (C) Growth assays for indicated strains at 37°C in SD low glucose medium (glucose- 0.1%). (D) Morphology of wild-type (WT) and ∆*hex1* strains injected into the peritoneal cavity of mouse. Peritoneal exudates were retrieved after 24 h of injection. *Candida albicans* cells of indicated strains engulfed by macrophages were stained with PAS stain. Arrows indicate *C. albicans* cells engulfed by macrophages. (E) 10 μl of peritoneal exudates of mice injected with indicated strains was added to 190 μL sterile MQ and plated on YPD agar plates to determine colony forming units. Error bars represent standard error between three biological replicates.

Although both growth and filamentation were normal in *Δhex1* strain under in vitro conditions, it was obvious for us to detect the behavior of mutant within the mammalian host. To examine this, we analyzed the virulence of *Δhex1* strain in mouse systemic candidiasis model. Wild-type and revertant strains were also included for comparison. Mice were injected with 1 × 10^7^ cells of each strain. In the group of mice injected with wild-type and revertant strain, all the animals died within 8.0 and 18 days, respectively, post injection (Fig.3A). However, group of mice injected with *Δhex1* strain, were more tolerant and 40% animals survived even after 45 days post injection. The result demonstrates that Hex1 significantly contributes to the virulence of the pathogen. Two mice from each group were sacrificed after 48 h of injection for histological examination of kidneys (Fig.3B). Kidney section of mice injected with wild-type strain showed rich growth of *C. albicans*. While no significant accumulation of pathogen was observed in the kidney sections of mice injected with *Δhex1* strain. To validate that reduced growth of *Δhex1* is not due to its inability to grow under low carbon source conditions, the growth was also analyzed in presence of 0.1% glucose. No significant difference was observed in the growth of *Δhex1* strain as compared to the wild-type or revertant strains in low glucose medium (Fig.3C). Thus, reduced growth of *∆hex1* strain in vivo could be due to inability to utilize available resources.

We also examined the behavior of *Δhex1* strain to the inflammatory response within the peritoneal cavity of the host. Wild-type cells and *Δhex1* strain were injected into the peritoneal cavity of mice. Cells were subsequently retrieved from the peritoneal exudates after 24 h of injection. A microscopic examination of the peritoneal exudates showed that the wild-type cells both engulfed by macrophage and nonengulfed showed filamentation, whereas only nonengulfed *∆hex1* mutant cells showed filamentation (Fig.3D). Most of the mutant cells were tightly packed within the macrophages showing only the yeast form. Survival of cells within the macrophages was determined by plating the exudates on YPD agar and counting the CFU. A 2.5-fold decrease in CFU was observed in *∆hex1* strain as compared to that of the wild type (Fig.3E).

### Role of *Hex1* in carbon scavenging

**Figure 4:**
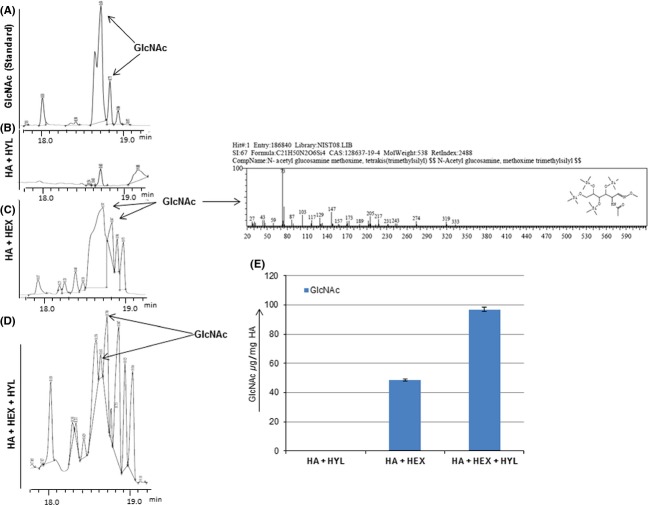
A section of chromatogram from GC/MS analysis of derivatized metabolites released upon HA breakdown. (A) Standard GlcNAc. (B) HA (0.4 mg/mL) + BTH (50 U/mL) treatment. (C) Purified Hex1 protein (50 U/mL) + (0.4 mg/mL) hyaluronic acid treatment. (D) Hex1 (50 U/mL) + (0.4 mg/mL) hyaluronic acid + BTH (50 U/mL) treatment. GC/MS analysis of pure GlcNAc was used as control. The average retention time of standard GlcNAc (*n* = 5) was observed to be 18.68 ± 0.05 min (GlcNAc methoxime derivative) and 18.77 ± 0.05 min (GlcNAc tetra-TMS derivative). Metabolites were identified from retention times and comparison of the corresponding mass spectrum with those in the NIST and Willey database. (E) Bar diagram represents the normalized concentration of GlcNAc identified in indicated sets of experiments. A total of 48.0 *μ*g GlcNAc was released per mg of HA in presence of hexosaminidase. In the presence of both hexosaminidase and hyaluronidase around 98.0 *μ*g of GlcNAc per mg HA was released. Error bars represent standard deviation within three biological replicates. GC/MS, gas chromatography-mass spectrometry; GlcNAc, *N*-acetyl-d-glucosamine; HA, hyaluronic acid; BTH, bovine testicular hyaluronidase; NIST, National Institute of Standards and Technology.

One of the possible roles of *β*-hexosaminidase could be scavenging of carbon due to its ability to hydrolyze the terminal nonreducing *β*-*N*-acetylglucosamine residues. GlcNAc has been shown to be a vital in vivo carbon source for the growth and virulence of several pathogens including *C. albicans*. In humans *β*-hexosaminidase is involved in the lysosomal degradation of HA. Hex1 was shown to be a secretory protein in *C. albicans* (Cannon et al. 1994). Thus, there is a possibility that *C. albicans* releases Hex1 in the ECM during infection and could obtain GlcNAc via breakdown of HA. We purified Hex1 from the culture media in presence of GlcNAc and confirmed it by Liquid chromatography-mass spectrometry (LC-MS) analysis (Fig. S3). The ability of purified CaHex1 to degrade HA (Rooster comb) was analyzed by an in vitro assay through estimation of GlcNAc released, using GC/MS analysis. Released GlcNAc was derivatized by both methoxyamine hydrochloride and MSTFA (Methods). Standard GlcNAc as determined by mass spectrometry showed two peaks at average retention time (RT) of (*n* = 5) 18.68 ± 0.05 min (GlcNAc methoxime derivative) and 18.77 ± 0.05 min (GlcNAc tetra-TMS derivative) (Fig.4A). In Hex1-treated samples, GlcNAc peaks were observed at RT of 18.70 and 18.80 min (Fig.4C) and in Hex1 and BTH-treated samples GlcNAc was observed at 18.65 and 18.73 min (Fig.4D). Difference observed within the RT of GlcNAc peaks was well within the average deviation in the RT of standard GlcNAc. GC/MS analysis showed that around 48 *μ*g GlcNAc was released per mg of HA in presence of Hex1 (Fig.4E). No significant release of GlcNAc was observed in presence of BTH (Fig.4B). This could be due to the fact that BTH is an endoglycosidase. However, in presence of both Hex1 and BTH, we observed around 98 *μ*g of GlcNAc per mg HA (Fig.4E). Thus, *C. albicans* Hex1 can efficiently degrade HA releasing free GlcNAc.

### Localization and accumulation of HA in kidneys

**Figure 5:**
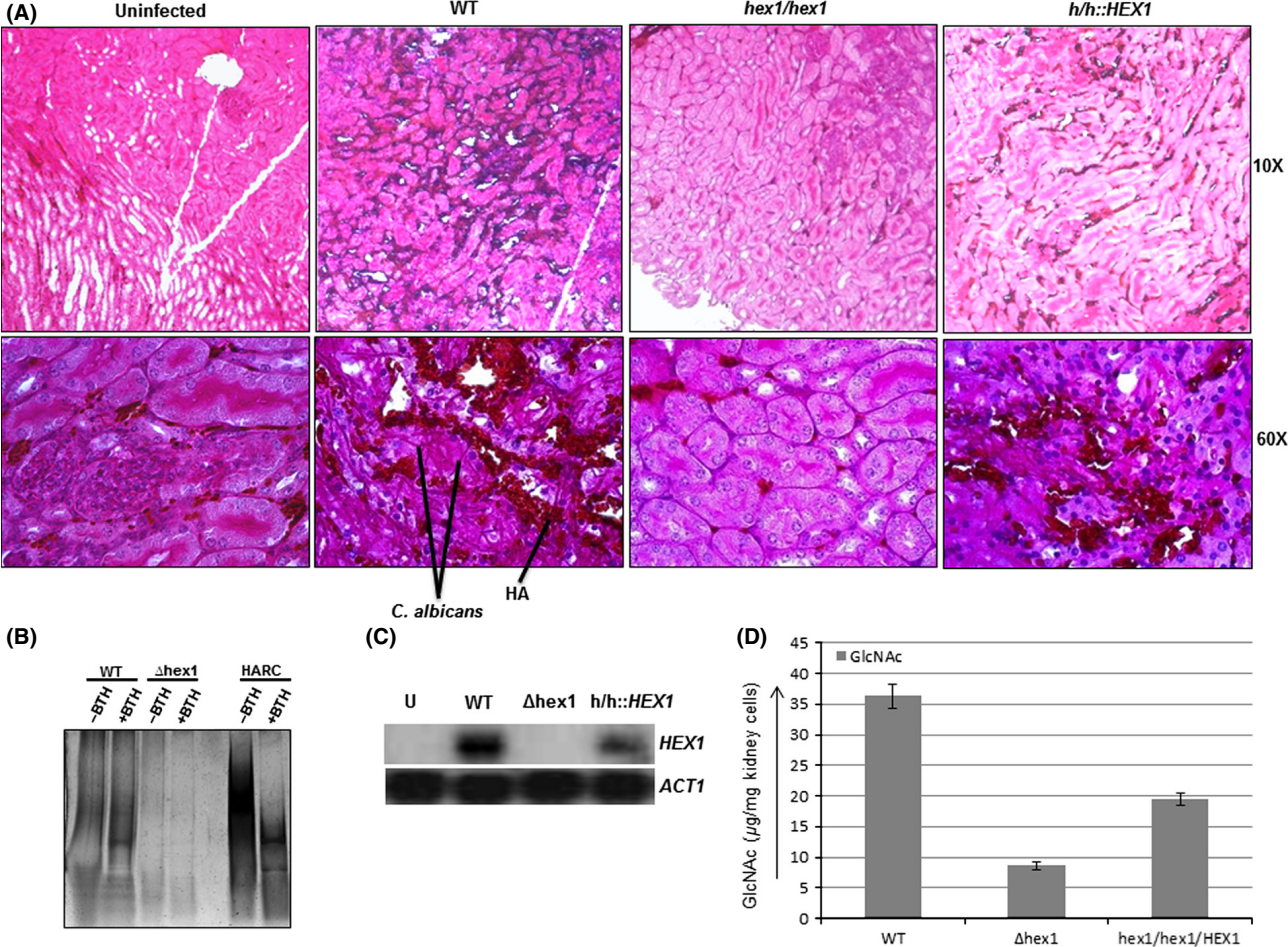
(A) Immunohistochemistry for detection of HA in kidneys of mice. HA was detected in kidney sections by staining with biotinylated HABP followed by treatment with Streptavidin-peroxidase and DAB. Sections were viewed at 10× and 60× magnifications. Significant accumulation of HA was detected in the kidneys of the mouse infected with the wild type and revertant strains. However, very little HA was observed in the uninfected and *Δhex1* infected mice kidneys. (B) Hyaluronan was purified from kidneys of mice infected with indicated stains of *Candida albicans* and then subjected to digestion with BTH (50 *μ*g/mL). Equal volumes of both the undigested and BTH-digested products from the kidneys were electrophoresed onto a 5–20% gradient polyacrylamide gel. Pure polymeric HA from rooster comb and HA treated with BTH were taken as controls. The gel was stained with 1.0% alcian blue in 3.0% acetic acid and then subsequently with silver nitrate. A higher level of polymeric HA in mice kidney infected with wild-type strain is clearly evident as compared with the mice kidney infected with *∆hex1* strain. (C) Northern blot analysis to check the expression of *C. albicans HEX1* in mice kidney infected with indicated strains. Equal loading was confirmed by methylene blue staining. Expression levels of mice *ACT1* was also determined in the blot. (D) Free GlcNAc in the kidney sections was assayed. Around 38 *μ*g of GlcNAc was present per mg tissue of kidney of mice infected with wild type strain SC5314. No significant release of GlcNAc was observed in *hex1* mutants. HA, hyaluronic acid; HABP, hyaluronic acid-binding protein assay; DAB, 3,3′-diaminobenzidine; BTH, bovine testicular hyaluronidase; GlcNAc, *N*-acetyl-d-glucosamine.

We also analyzed the localization and accumulation of HA in kidneys, since they are the primary colonization sites of *C. albicans* during candidiasis. Immunohistochemistry analysis showed a significant accumulation of HA in the kidneys of the mouse infected with the wild-type strain (Fig.5A). Similarly, the revertant strain also showed accumulation of HA in the kidneys but less than the kidneys infected with the wild-type strain. However, very little HA was observed in the mice kidney infected with the *Δhex1* strain. HA was also purified from the kidney samples (100 mg) and subjected to 5–20% gradient polyacrylamide gel electrophoresis (Fig.5B). For comparison, commercially available HA (rooster comb) was also subjected to electrophoresis. Higher amount of HA was observed in the kidney infected with wild-type strain as compared to *∆hex* strain. Higher accumulation of HA was also evident from BTH treatment, where high levels of polymeric HA from the mice kidney infected with wild-type strain, disappears with the appearance of increased levels of oligomeric HA. Expression of *C. albicans HEX1* was also analyzed in mice kidneys infected with wild-type and hex1 mutants (Fig.5C). Northern blot analysis showed significant expression of *C. albicans HEX1* in the kidney of mouse infected with wild-type strain. No expression of *HEX1* was observed in the kidneys of mouse infected with the *∆hex1* strain and uninfected kidneys. Around twofold less expression of *HEX1* was observed in the *hex1/hex1/HEX1*-infected kidneys. Free GlcNAc in kidneys of mice infected with wild-type, *∆hex1*, and *hex1/hex1/HEX1* strains were also compared (Fig.5D). Estimation of GlcNAc using dimethylaminobenzaldehyde reagent (Elson and Morgan 1933) showed five times higher amount of free GlcNAc in the kidneys of mice infected with wild-type strain as compared to the kidneys infected with *∆hex1* strain. Free GlcNAc in kidneys of mice infected with *hex1/hex1/HEX1* revertant strain was intermediate to the levels of kidneys of mice infected with wild-type and *∆hex1*strains.

## Discussion

GlcNAc is a monosaccharide derivative of glucose and an abundant hexose that plays diverse role in several biological systems. Within the cells O-GlcNAc posttranslational modifications is required to control the activity of many different cytoplasmic and nuclear proteins. Prominent examples where GlcNAc is required for intracellular signaling is the regulation of insulin pathway (Slawson and Hart 2003; Slawson et al. 2006), proteosome function (Zachara and Hart 2004), critical transcription factors like c-myc and p53 (Chou and Hart 2001; Yang et al. 2006). GlcNAc also contributes to the structure and function of various ECMs. In addition to this GlcNAc is part of membrane-inserted sugar lipid complexes (Eisenhaber et al. 2003; Haltiwanger and Lowe 2004). GlcNAc is also a precursor of *N*-acetylated sugars and sialic acid (Tanner 2005). Lately, it has been shown that GlcNAc can be used as in vivo source of energy by human pathogens. One of the potential sources of GlcNAc within the human body is HA present in the ECM. In this study, we have characterized *β*-hexosaminidase gene (*HEX1*) in *C. albicans* and explored its putative function as GlcNAc scavenger via breakdown of HA.

Earlier it was reported that *HEX1* could be an essential gene in *C. albicans*. Homozygote trisome test showed that it is a nonessential gene and subsequently double allele knockout of *HEX1* was generated by SAT flipper technique. Virulence analysis in mice showed that *∆hex1* mutant was significantly less virulent as compared to the wild-type and revertant strains. Response of *∆hex1* strain to macrophages within the peritoneal cavity was also analyzed. Interestingly, as compared to wild-type strain more *∆hex1* cells were phagocytosed by the macrophages. Survival of *∆hex1* cells was 2.5-fold less as compared to wild type in response to macrophages. *∆hex1* cells were observed to be densely packed within the macrophages and were locked in yeast form. Moreover, *∆hex1* cells not phagocytosed by the macrophages showed hyphal form. Thus, both survival and morphogenesis were affected in response to phagocytes of the innate immune system. Although, it was earlier shown that glycolytic genes are not induced following phagocytosis by macrophages (Barelle et al. 2006). Previously it was also reported that GlcNAc metabolic pathway genes (Lorenz et al. 2004) and GlcNAc transporter (Alvarez and Konopka 2007) are induced upon phagocytosis by macrophages. So, further studies are required to show how induction of genes by GlcNAc is required for countering the macrophages which is the first line of defence against this pathogen.

*β*-hexosaminidase enzyme was purified from the culture medium and in vitro assay showed that it has the ability to digest HA releasing free GlcNAc. It was also observed that in presence of hyaluronidase, twofold higher GlcNAc was released as compared to Hex1 alone. Thus, presence of hyaluronidase in *C. albicans* was also explored. Earlier it was reported that *C. albicans* can produce hyaluronidase (Shimizu et al. 1995). But BLAST analysis in Candida genome database with known hyaluronidase did not show any significant hit. Moreover, mucopolysaccharase activity analysis on plates supplemented with HA also showed absence of hyaluronidase activity in *C. albicans* (data not shown). HA distribution in kidneys was analyzed by immunohistochemistry as it is among the main organ involved in Candidiasis disease. Examinations of kidney section of the mouse infected with *C. albicans* showed sever accumulation of HA in the renal cortex. The accumulation of HA increased in response to higher level of *C. albicans* infection. In uninfected kidney, negligible amount of HA was observed in the cortex. It is known that renal injury induces the release of cytokines, which can induce fibroblast and tubular epithelial cells to increase HA expression (Heldin et al. 1989; Feusi et al. 1999). Thus, secretion of excessive amount of HA could be response of renal injury caused by *C. albicans* infection. Northern blot analysis showed that *HEX1* is expressed during kidney infection. Significant amount of GlcNAc was also detected the wild-type infected kidney, which could be a result of HA breakdown by *C. albicans* Hex1 and mammalian hyaluronidase. It was earlier believed that GlcNAc is required for skin and mucosal infections of *C. albicans* but not required for systemic infections. Presence of HA in kidneys in response to Candida infection showed that organ colonization of this pathogen could be dependent on GlcNAc.

## Supporting Information

**Figure S1.** Phylogenetic relationships of Hex1 proteins of various species. The sequences were aligned by CLUSTALW in MEGA6 program and the unrooted phylogenetic tree was constructed by Neighbor-Joining method with 1000 bootstrap replicates. The evolutionary distances were computed using p-distance method. The bootstrap values are shown at the nodes. Accession numbers of Hex1 of each species are provided in brackets. **Figure S2.** Southern blot analysis to confirm the null mutant of *HEX1* by SAT Flipper. (A) Autoradiogram representing the first allele knockout of *HEX1*. Strains with a 4.5 and 1.9 kb band are 1st allele knockouts and wild-type strain with 1.9 kb band was used as control. (B) Autoradiogram to confirm the null mutant of *HEX1*. *Represents the double allele knockout strain. L, 1Kb ladder; U, undigested DNA; C, SC5314; Hh1, first allele knockout. 0.5 kb upstream region of *HEX1* was used as probe. (C) Autoradiogram representing reintegration of *HEX1* allele into ∆hex1 strain. Strains showing 6.6 kb band were revertant hex1/hex1/*HEX1* while the Δhex strain showed only 0.8 and 0.3 kb bands. For Southern blot DNA digested with *BglII* enzyme and 0.5 kb upstream region of *HEX1* was used as probe for hybridization. **Figure S3.** (A) *β*-hexosaminidase enzyme activity. One unit of enzyme was defined as that which catalyzed the formation of 1 µmol of p-nitrophenol per min. (B) SDS PAGE analysis of proteins secreted into the culture media. Cells were grown in indicated media and the culture media was concentrated using centricons (3 kDa). Protein was precipitated with chilled acetone and the pellet was air dried and redissolved in 1X PBS. About 25 µg protein was loaded in each well and stained with silver stain. Arrow indicates differential band (∼115 kDa). (C) SDS-PAGE analysis of fraction obtained after gel filtration chromatography having highest hexosaminidase activity. Gel was stained with silver stain. (D) Graph showing molecular weight determination of *CaHEX1* by gel filtration chromatography. *CaHEX1* was eluted at elution volume of 13.184 (Kav −0.324) which corresponds to a mol wt of 103.7 kDa after comparison with standards of protein. Molecular weight markers used as standards were Catalase (232 kDa), Aldolase (158 kDa), Albumin (67 kDa), Ovalbumin (43 kDa), and Chymotrypsin (25 kDa).
