# Supplementary figures and images for: In vivo role of Candida albicans β-hexosaminidase (HEX1) in carbon scavenging
Source: Microbiologyopen. 2015 Jul 14;4(5):730–42. doi: 10.1002/mbo3.274 (PMC4618606; doi:10.1002/mbo3.274)

Supplementary Figure S1

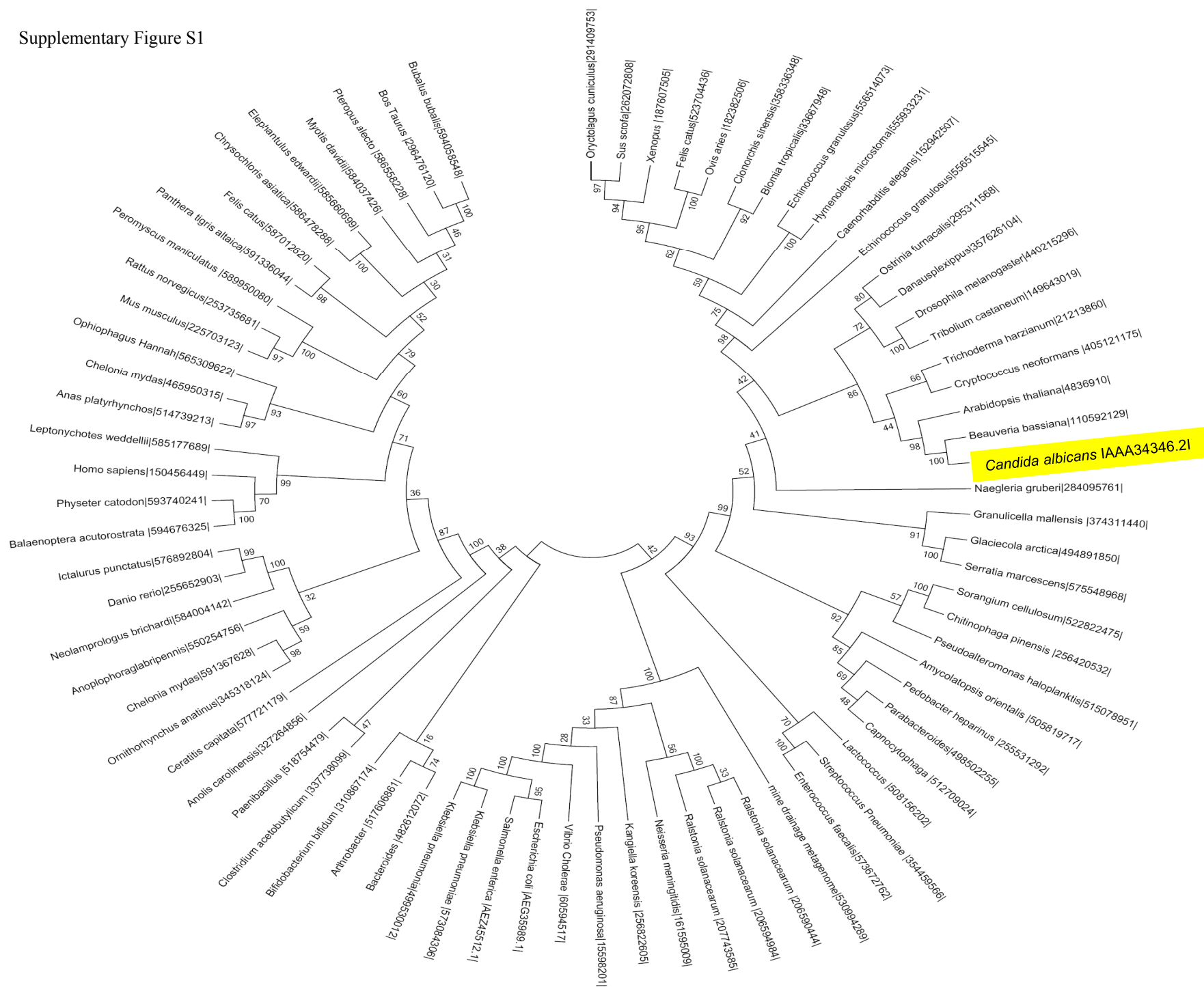

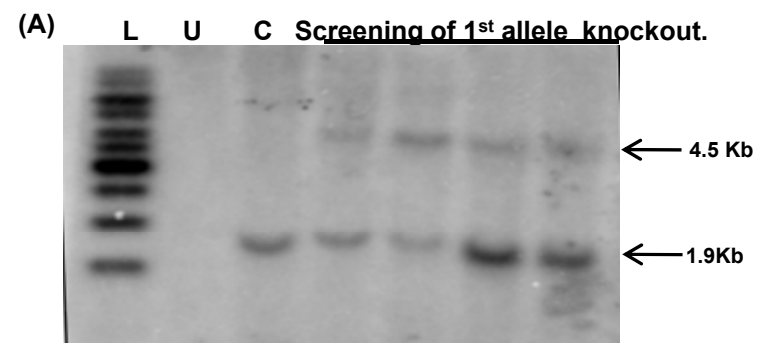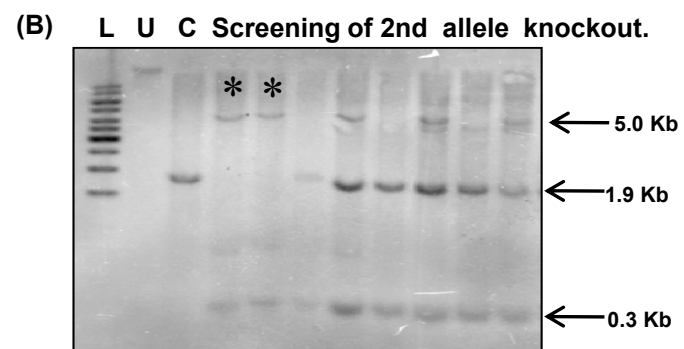

Supplementary Figure S3

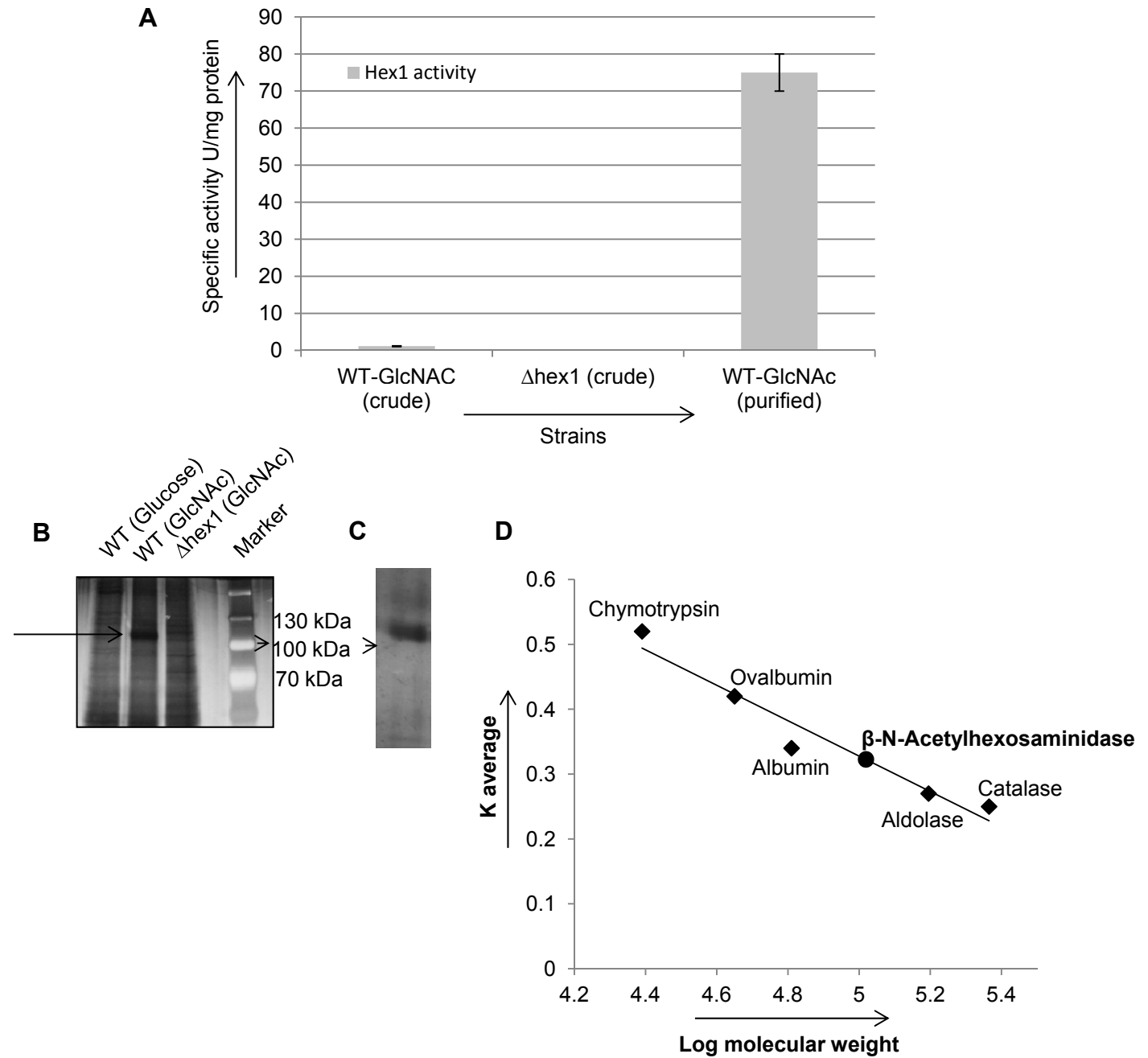

Supplement: Supplementary file 1 — Figure S1. Phylogenetic relationships of Hex1 proteins of various species. The sequences were aligned by CLUSTALW in MEGA6 program and the unrooted phylogenetic tree was constructed by Neighbor-Joining method with 1000 bootstrap replicates. The evolutionary distances were computed using p-distance method. The bootstrap values are shown at the nodes. Accession numbers of Hex1 of each species are provided in brackets. Figure S2. Southern blot analysis to confirm the null mutant of HEX1 by SAT Flipper. (A) Autoradiogram representing the first allele knockout of HEX1. Strains with a 4.5 and 1.9 kb band are 1st allele knockouts and wild-type strain with 1.9 kb band was used as control. (B) Autoradiogram to confirm the null mutant of HEX1. *Represents the double allele knockout strain. L, 1Kb ladder; U, undigested DNA; C, SC5314; Hh1, first allele knockout. 0.5 kb upstream region of HEX1 was used as probe. (C) Autoradiogram representing reintegration of HEX1 allele into ∆hex1 strain. Strains showing 6.6 kb band were revertant hex1/hex1/HEX1 while the Δhex strain showed only 0.8 and 0.3 kb bands. For Southern blot DNA digested with BglII enzyme and 0.5 kb upstream region of HEX1 was used as probe for hybridization. Figure S3. (A) β-hexosaminidase enzyme activity. One unit of enzyme was defined as that which catalyzed the formation of 1 µmol of p-nitrophenol per min. (B) SDS PAGE analysis of proteins secreted into the culture media. Cells were grown in indicated media and the culture media was concentrated using centricons (3 kDa). Protein was precipitated with chilled acetone and the pellet was air dried and redissolved in 1X PBS. About 25 µg protein was loaded in each well and stained with silver stain. Arrow indicates differential band (∼115 kDa). (C) SDS-PAGE analysis of fraction obtained after gel filtration chromatography having highest hexosaminidase activity. Gel was stained with silver stain. (D) Graph showing molecular weight determination of CaHEX1 by gel filtrat [file mbo30004-0730-sd1.pdf]
