# Supplementary material for: In vivo role of Candida albicans β-hexosaminidase (HEX1) in carbon scavenging
Source: Microbiologyopen. 2015 Jul 14;4(5):730–42. doi: 10.1002/mbo3.274 (PMC4618606; doi:10.1002/mbo3.274)
Supplement: Supplementary file 2 — Data S1. Methods. [file mbo30004-0730-sd2.doc]

**Supplementry Information**

**Methods:**

***SAT Flipper based gene disruption***

SAT-Flipper Strategy based on Nourseothricin antibiotic selection was used to make a null mutant of *CaHEX1* gene. Nourseothricin is a member of the streptothricin group of antibiotics produced by *Streptomyces noursei.* The *SAT1* gene from the bacterial transposon Tn1825 encodes streptothricin acetyltransferase, which confers resistance to nourseothricin by inactivating the antibiotic. A *HEX*1 deletion cassette was constructed in the following way: A *KpnI*–*XhoI* fragment of the *C. albicans* *HEX1*gene (positions -500 to +10) was amplified from SC5314 genomic DNA with the primer *HF1* and *HR1* (5̕-TCAGAAggtaccGAAATAATACAACATAAT-3̕ and 5̕-GAAAGActcgagTTTATCTA ACACCATTTTG-3̕) and a *PstI*–*SacI* downstream fragment from positions + 2689 to + 3220 was amplified with the primer HF2 and HR2 (5̕-AAAATCctgcagTTTATTGAATGT ATAGAGAT-3̕ and 5̕-ACAGAAAgagctcCACGACTATGTCCCT-3̕). Nucleotide positions are with respect to the start codon of the *HEX1*gene. The *HEX1* upstream and downstream fragments were cloned on both sides of the SAT1 flipper to generate *HEX1* disruption cassette (*HEX1/::FRT:SAT1-FLIP::FRT::HEX1*) and the plasmid was named as pHSATEX. The complete deletion cassette was excised from the plasmid by *KpnI* and *SacI* digestion. The cassette was transformed in *C. albicans* by electroporation. Homologous recombination would replace the *HEX1* coding region from position -10 to + 2689 (directly before the stop codon) by the SAT1 flipper. After transformation, cells were spread on an YPD plate containing 200 µg/mL Nourseothricin. Nourseothricin-resistant colonies were picked and streaked onto a new YPD plate with 200 µg/mL Nourseothricin. DNA of the transformants was digested with *BglII* restriction endonuclease for Southern blot analysis to identify first allele knockouts (Fig. S2A). The heterozygous mutants were then cured for SAT marker for second round of transformation to get null mutants. Correct excision of the SAT1 flipper cassette in Nourseothricin-sensitive clones (small colonies selected in presence of 20 µg/mL Nourseothricin) was confirmed by Southern blot analysis. One correct Nourseothricin-sensitive derivative was then used for a second round of integration/excision of the SAT1 flipper cassette to obtain homozygous null mutants (Fig. S2B). The second allele disruptants were confirmed by southern analysis as described earlier. Clone with 4.9kb and 0.3kb bands were double allele knockouts (*∆hex1*). A αP^32^ labeled 0.5kb upstream region of *HEX1* was used as probe for Southern hybridizations.

***Generation of Revertant***

For reintegration of *HEX1*gene into the original loci of homozygous *hex1* mutant, *ApaI*–*SalI* fragments containing the complete open reading frame as well as 0.5kb of upstream and 0.44 kb of downstream flanking sequences of the *HEX1* was amplified with the primers HF2 (5̕-CGGGGTACC TGAATGTCAAGACTGTTGTCC- 3̕) and HR2 (5̕-ATATAGTCGACAGATCTATAAACATCAC GTTTCTCTCCG-3̕) and cloned into *ApaI-SalI* digested pHSATEX plasmid to generate pHEXRV plasmid. Disruption cassette was removed by *KpnI* and *SacI* digestion and transformed into Δhex strain by electroporation. For confirming the reintegration Southern blot analysis was performed (Fig. S2C). DNA of the transformants was digested with *BglII* enzyme. A αP^32^ labeled 0.5kb upstream region of *HEX1* was used as probe for Southern hybridizations.

***β-N-acetylglucosaminidase enzyme assay.***

β-*N*-acetylglucosaminidase enzyme assay was performed as described elsewhere (Sullivan *et al*., 1984), using artificial substrate 4-Nitrophenyl N-acetyl- β-D-glucosaminide (pNP). One unit of enzyme was defined as that which catalyzed the formation of 1 µmol of p-nitrophenol per min.

***Purification of hexosaminidase protein.***

Overnight grown preculture of SC5314 (wild type) in YPD medium was diluted 1:100 times in SD and grown till mid log phase. Cells were harvested, washed twice with 0.3% KH_2_PO_4_, and was resuspended in SN medium. The cultures were grown at 37°C at 200 rpm for 4 hrs. Cells were removed from the medium by centrifugation at 4000rpm at 4°C for 15minutes. Medium was then concentrated in Millipore centricon of 3 kDa cutoff molecular weight from 200 ml supernatant to 5 ml and then gel filtration chromatography was performed on superdex 200 gel filtration column. The column was equilibrated with 20 mM Tris-Cl, pH 7.0 and 200 mM NaCl. Fractions were collected after the void volume, assayed for enzyme activity and resolved on 12.5% SDS –PAGE. The enzyme rich fractions were concentrated using Centricon (3 kDa cut off) and stored at 4^o^C and sample assayed for β-D-N-acetylhexosaminidase activity.

***Molecular size determination***

Native molecular weight of β-D-N-acetylhexosaminidase was determined on superdex G-200 analytical column. The column was equilibrated with 20mM Tris-Cl, pH 7.0 and 200 mM NaCl. Further the column was calibrated with standard proteins of known molecular weight like, Thyroglobulin (669 kDa), Ferritin (440 kDa), Catalase (232 kDa), Lactase dehydrogenase (140 kDa) and Bovine serum albumin (67 kDa). Void volume of the column was determined by running blue dextran through the column. Then 100ul of unknown protein (~100 ug) was loaded on the column and run with same flow rate as that of the standards. Protein standards as well as the samples were run in duplicates and average value (elution volume) was taken for the calculations. Calibration curve for molecular weight was drawn by plotting Kav values against log molecular weights. Kav was calculated using the formula:

Kav =Ve-Vo/Vt-Vo.

Ve = Elution volume of the protein; Vo = Void volume (elution volume of the blue dextran);

Vt = Total bed volume.

**Results**

*Purification of Hex1*

Hex1 is predicted to be a secretory protein due to presence of signal peptide. To verify this, we estimated the Hex1 activity in the GlcNAc containing culture media and obtained the specific activity of 1.15 U/mg protein after 4hrs of growth of *C. albicans* at 37⁰C (Fig. S3A). The proteins in the culture media were concentrated by centricon (3 kDa, Millipore) followed by acetone precipitation. The precipitated protein was subjected to SDS gel electrophoresis. As compared to glucose media we observed a prominent differential band (~115 kDa) in the GlcNAc media (Fig. S3B). This band was absent in the GlcNAc culture media of *∆hex1* strain. Protein was further purified by gel filtration chromatography. Fractions with β-hexosaminidase activity were pooled and concentrated by centricons (3 kDa cut off). SDS PAGE analysis of this pure fraction again showed a band of 115 kDa (Fig. S3C). Although the expected molecular weight of Hex1 is ≈ 63.5 kDa, but a high molecular weight band of ≈ 115 kDa is observed in SDS PAGE analysis due to highly glycosylated nature of hex1protein (Cannon *et al*., 1994). The band was excised and identified as Hex1by LC-MS analysis. In MS/MS analysis 8 peptides matched to the Hex1 protein with 9 percent coverage area giving a score of 198. Through gel filtration, Hex1 peak was observed at elution volume of 13.184 (Kav- 0.324) which corresponds to a molecular weight of ≈103.7 kDa (Fig. S3D).

*Supplementary Figure Legends*

S1*-* **Phylogenetic relationships of Hex1 proteins of various species.** The sequences were aligned by CLUSTALW in MEGA6 program and the unrooted phylogenetic tree was constructed by Neighbor-Joining method with 1000 bootstrap replicates. The evolutionary distances were computed using p-distance method. The bootstrap values are shown at the nodes. Accession numbers of Hex1 of each species is provided in brackets.

S2- **Southern blot analysis to confirm the null mutant of *HEX1* by SAT Flipper. (**A) Autoradiogram representing the 1^st^ allele knockout of *HEX1.* Strains with a 4.5Kb and 1.9 Kb band are 1^st^ allele knockouts and wild type strain with 1.9 Kb band was used as control. (B) Autoradiogram to confirm the null mutant of *HEX1.* * represents the double allele knockout strain. L, 1Kb ladder; U, undigested DNA; C, SC5314; Hh1, 1^st^ allele knockout. 0.5kb upstream region of *HEX1* was used as probe. (C) Autoradiogram representing reintegration of *HEX1* allele into ∆hex1 strain. Strains showing 6.6Kb band were revertant hex1/hex1/*HEX1* while the Δhex strain showed only 0.8kb and 0.3kb bands. For Southern blot DNA digested with *BglII* enzyme and 0.5kb upstream region of *HEX1* was used as probe for hybridization.

**S3**- A) β-hexosaminidase enzyme activity. One unit of enzyme was defined as that which catalyzed the formation of 1 µmol of p-nitrophenol per min. B) SDS PAGE analysis of proteins secreted into the culture media. Cells were grown in indicated media and the culture media was concentrated using centricons (3 kDa). Protein was precipitated with chilled acetone and the pellet was air dried and redissolved in 1XPBS. 25 µg protein was loaded in each well and stained with silver stain. Arrow indicates differential band (~115 kDa). C) SDS-PAGE analysis of fraction obtained after gel filtration chromatography having highest hexosaminidase activity. Gel was stained with silver stain. D) Graph showing molecular weight determination of *CaHEX1* by gel filtration chromatography. *CaHEX1* was eluted at elution volume of 13.184 (Kav- 0.324) which corresponds to a mol wt of 103.7 kDa after comparison with standards of protein. Molecular weight markers used as standards were Catalase (232 kDa), Aldolase (158 kDa), Albumin (67 kDa), Ovalbumin (43 kDa) and Chymotrypsin (25 kDa).
